# Molecular, Morphological, and Functional Characterization of Corticotropin-Releasing Factor Receptor 1-Expressing Neurons in the Central Nucleus of the Amygdala

**DOI:** 10.1523/ENEURO.0087-19.2019

**Published:** 2019-06-17

**Authors:** S. A. Wolfe, H. Sidhu, R. R. Patel, M. Kreifeldt, S. R. D’Ambrosio, C. Contet, M. Roberto

**Affiliations:** Department of Neuroscience, The Scripps Research Institute, La Jolla, CA 92037

**Keywords:** calcium binding proteins, corticotropin-releasing factor, dendritic spines, glutamatergic signaling, neuropeptides, stress and anxiety

## Abstract

The central nucleus of the amygdala (CeA) is a brain region implicated in anxiety, stress-related disorders and the reinforcing effects of drugs of abuse. Corticotropin-releasing factor (CRF, *Crh)* acting at cognate type 1 receptors (CRF_1_, *Crhr1*) modulates inhibitory and excitatory synaptic transmission in the CeA. Here, we used CRF_1_:GFP reporter mice to characterize the morphological, neurochemical and electrophysiological properties of CRF_1_-expressing (CRF_1_+) and CRF_1_-non-expressing (CRF_1_–) neurons in the CeA. We assessed these two neuronal populations for distinctions in the expression of GABAergic subpopulation markers and neuropeptides, dendritic spine density and morphology, and excitatory transmission. We observed that CeA CRF_1_+ neurons are GABAergic but do not segregate with calbindin (CB), calretinin (CR), parvalbumin (PV), or protein kinase C-δ (PKCδ). Among the neuropeptides analyzed, *Penk* and *Sst* had the highest percentage of co-expression with *Crhr1* in both the medial and lateral CeA subdivisions. Additionally, CeA CRF_1_+ neurons had a lower density of dendritic spines, which was offset by a higher proportion of mature spines compared to neighboring CRF_1_– neurons. Accordingly, there was no difference in basal spontaneous glutamatergic transmission between the two populations. Application of CRF increased overall vesicular glutamate release onto both CRF_1_+ and CRF_1_– neurons and does not affect amplitude or kinetics of EPSCs in either population. These novel data highlight important differences in the neurochemical make-up and morphology of CRF_1_+ compared to CRF_1_– neurons, which may have important implications for the transduction of CRF signaling in the CeA.

## Significance Statement

Corticotropin-releasing factor (CRF) is involved in emotional regulation via hypothalamic and amygdalar circuits, and is implicated in several psychiatric disorders including anxiety, depression, addiction, posttraumatic stress disorder, and eating disorders. Our novel identification of specific molecular, morphologic and functional properties to distinguishing CRF receptor type 1 (CRF_1_)+ neurons in the central amygdala represents a critical step in understanding the cellular role and dysregulation of the CRF system in pathologic conditions.

## Introduction

The corticotropin-releasing factor (CRF, *Crh*) plays an important role in emotional regulation via hypothalamic and amygdalar circuits under normal physiologic conditions (Heilig et al., 1994). Accordingly, dysregulation of the CRF system is implicated in several animal models of psychiatric disorders. Despite promising preclinical studies in animal models including anxiety, depression, addiction, posttraumatic stress disorder, and eating disorders (Menzaghi et al., 1994; Koob, 2003, 2008; Valdez et al., 2003; Chu et al., 2007; Funk et al., 2007; Ciocchi et al., 2010; Bruijnzeel et al., 2012; Iemolo et al., 2013; Ji et al., 2013; Baiamonte et al., 2014; Tye et al., 2011). The clinical studies of CRF receptor type 1 (CRF_1_) antagonists on mood disorders have been unsuccessful (Zorrilla and Koob, 2004; Nielsen, 2006; Heilig et al., 2011; Kwako et al., 2015; Schwandt et al., 2016; Dunlop et al., 2017). It has been suggested that these differences may be due to several factors including differences between human symptomatology compared to animal behavior, differences in contributions of different CRF_1_ populations, or functional state of CRF_1_ between humans and animals, and dosage and bioavailability of the CRF_1_ antagonists (Zorrilla and Koob, 2004; Nielsen, 2006; Binneman et al., 2008; Kehne and Cain, 2010; Cruces et al., 2014; Dong et al., 2018). Despite these pitfalls, further research is needed to understand more mechanistically the CRF/CRF_1_ system which could develop CRF_1_ antagonists to be suitable for patient treatment with improved bioavailability and decreased side-effects in humans (Pomrenze et al., 2017; Spierling and Zorrilla, 2017; Dong et al., 2018).

CRF and CRF_1_ (*Crhr1*) expressing neurons are located in several brain regions including the central nucleus of the amygdala (CeA), which functions as the main output nucleus for amygdala functions (Gilpin et al., 2015). The CeA is comprised of medial (CeM) and lateral (CeL) subdivisions (Sun et al., 1991; Petrovich et al., 1996; Dong et al., 2001; Ciocchi et al., 2010; Davis et al., 2010; Haubensak et al., 2010; McCullough et al., 2018) and contains mostly GABAergic projection neurons and interneurons (Sun and Cassell, 1993; Veinante and Freund-Mercier, 1998). The CeM sends inhibitory projections to various effector regions [e.g., hypothalamus, periaqueductal gray, locus coeruleus, bed nucleus of the stria terminalis (BNST), and pedunculopontine tegmental nucleus; Pitkänen and Amaral, 1994]. The CeL sends inhibitory inputs to CeM, thereby gating the output activity of the CeA, but also to more distant brain regions such as the periaqueductal gray and paraventricular nucleus of the thalamus (Penzo et al., 2014).

Accumulating evidence implicates the CRF/CRF_1_ system in the CeA in many animal models of physiological and pathological conditions (Chu et al., 2007; Funk et al., 2007; Roberto et al., 2010; Bruijnzeel et al., 2012; Lowery-Gionta et al., 2012; Iemolo et al., 2013; Ji et al., 2013; Baiamonte et al., 2014). However, cellular heterogeneity in this region has limited the identification and full functional characterization of the CRF_1_-expressing (CRF_1_+) subset. Thus, we used a bacterial artificial chromosome (BAC) transgenic mouse line expressing the green fluorescent protein (GFP) under the *Crhr1* promoter (CRF_1_:GFP) to readily identify neurons expressing CRF_1_ (Justice et al., 2008). Previously CRF_1_+ neurons were observed to be mainly located in the CeM and exhibit an ongoing tonic GABAergic conductance driven by action potential-dependent GABA release. In contrast, CRF_1_ non-expressing (CRF_1_–) neurons display no ongoing tonic inhibition (Herman et al., 2013). Although this functional analysis has yielded significant insight into cell type-specific properties in CeA microcircuits, a precise molecular characterization of the CRF_1_+ neuronal population is lacking.

The expression pattern of several relevant markers in the rat and mouse CeA have been used to distinguish neuronal subsets including: calcium-binding proteins (CBPs; Andressen et al., 1993; Kemppainen and Pitkänen, 2000), neuropeptides such as CRF (Petrovich et al., 1996), somatostatin (*Sst*; Li et al., 2013; Penzo et al., 2014; Yu et al., 2016; Kim et al., 2017), proenkephalin (PENK, *Penk*; Poulin et al., 2008), prodynorphin (PDYN, *Pdyn*; Merchenthaler et al., 1997; Funk et al., 2006; Schwarzer, 2009), neuropeptide Y (NPY, *Npy*; Lin et al., 2006; McGuire et al., 2011; Gilpin et al., 2015), and protein kinase C-δ (PKC-δ, *Prkcd*; Herry et al., 2008; Haubensak et al., 2010; Cai et al., 2014). However, the patterns of co-expression of these markers with *Crhr1* are unknown.

Given the critical role of CRF and CRF_1_ in the CeA, we sought to identify unique molecular, morphologic and functional properties that distinguish CeA CRF_1_+ neurons from their CRF_1_– neighbors. Utilizing CRF_1_:GFP mice we determined the following characteristics of CeA CRF_1_+ and CRF_1_– neurons: (1) co-expression patterns of CRF_1_ (via GFP reporter) with CBPs parvalbumin (PV), calretinin (CR), and calbindin (CB); (2) co-expression patterns of *Crhr1* with *Penk*, *Pdyn*, *Sst*, *Npy*, *Crh*, and *Prkcd*; (3) dendritic spine morphology and density; and (4) basal and CRF-modulated glutamatergic transmission. We found that *Penk* and *Sst* have the highest percentage of co-expression with *Crhr1* in both the CeM and CeL, and that *Penk* is enriched in CeM *Crhr1*+ neurons compared to their *Crhr1*– neighbors. We also show that CeM CRF_1_+ neurons exhibit an overall lower spine density and differential fractions of mature versus immature spines compared to CRF_1_– neurons. Consistent with a comparable density of mature spines in these two populations, we found no difference in spontaneous EPSCs (sEPSCs) or miniature EPSCs (mEPSCs). Importantly, acute CRF application increased overall CeM glutamatergic transmission and does not affect amplitude or kinetics of EPSCs in either population. These data provide important information about the neurobiology of CeA CRF_1_+ neurons that may have critical implications for their functional role under physiological and pathological conditions.

## Materials and Methods

All procedures were approved by our Institutional Animal Care and Use Committee and were consistent with the National Institutes of Health Guide for the Care and Use of Laboratory Animals. Transgenic CRF_1_:GFP mice were generated on a mixed C57BL/6J x BALB/c background using BAC recombination (see Justice et al., 2008 for transgene design and immunohistochemical validation of reporter expression). A colony was established at our institute in 2008 and has been backcrossed to C57BL/6J mice every two to three years since. Mice were genotyped by PCR on tail snip lysates using the following transgene-specific primers: forward 5'-GGT CAC CCC AAA AAT AAT CTC T-3'; reverse 5'-AGG ATT GGG AAG ACA ATA GC-3'. We also amplified a positive control band using the following primers: forward 5'-TCC TCA AAG ATG CTC ATT AG-3'; reverse 5'-GTA ACT CAC TCA TGC AAA GT-3'. Adult male mice were used for all experiments.

### Immunohistochemistry

#### Tissue preparation

Mice (*n* = 4) were anesthetized (isoflurane) and perfused with cold PBS followed by 4% paraformaldehyde (PFA). Brains were dissected and immersion fixed in PFA for 24 h at 4°C, cryoprotected in sterile 30% sucrose in PBS for 24–48 h at 4°C or until brains sank, flash frozen in pre-chilled isopentane on dry ice, and stored at –80°C. Free floating 35-µm brain sections were obtained using a cryostat and kept at 4°C in PBS containing 0.01% sodium azide.

#### Immunohistochemistry

Sections were washed in PBS for 10 min at room temperature (RT) with gentle agitation and then blocked for 1 h at RT in blocking solution [0.3% Triton X-100, 1 mg/ml bovine serum albumin (BSA), and 5% normal goat serum (NGS)]. Primary antibody was incubated at 4^0^C overnight with gentle agitation in 0.5% Tween 20 and 5% NGS. The following primary antibodies were used: chicken anti-GFP (Abcam, ab13970, RRID:AB_300798; 1:2000), mouse anti-PV (Swant, 235, RRID:AB_10000343; 1:1000), mouse anti-CR (Swant, 6B3, RRID:AB_10000320; 1:500), and mouse anti-CB (Swant, 300, RRID:AB_10000347; 1:2000). Next, sections were triple washed in PBS for 10 min at RT with gentle agitation followed by a 1-h secondary antibody incubation in PBS (in the dark). The following secondary antibodies were used: Alexa Fluor 488 goat anti-chicken (Thermo Fisher Scientific, A-11039, RRID:AB_142924) and Alexa Fluor 568 goat anti-mouse (Thermo Fisher Scientific, A-11004, RRID:AB_141371). Sections were then washed (10 min, RT, three times) and mounted in Vectashield (Vector labs, H1500, RRID:AB_2336788).

#### Imaging and analysis

Sections were imaged on a Zeiss LSM 780 laser scanning confocal microscope (10× objective, tile scanning of CeA). All microscope settings were kept the same within experiments during image acquisition. Analyst was blind to the identity of the red fluorescent signal (CBP) when performing cell counts, and analysis was performed manually in an unbiased manner at four anterior-posterior levels (equidistant sections located –1.00 through –1.70 mm from bregma). Data are presented as mean ± SEM.

### *In situ* hybridization

#### Tissue preparation

Mice (*n* = 3–4) were anesthetized (isoflurane) and perfused with cold PBS/Z-fix (Fisher Scientific, NC9378601). Brains were dissected and immersion fixed in Z-fix for 24 h at 4°C, cryoprotected in sterile 30% sucrose in PBS for 24–48 h at 4°C or until brains sank, flash frozen in pre-chilled isopentane on dry ice, and stored at –80°C. Brains were then sliced on a cryostat in 20-µm-thick sections, mounted on SuperFrost Plus slides (Fisher Scientific, 1255015) and stored at –80°C.

#### *In situ* hybridization

*In situ* hybridization was performed using RNAscope fluorescent multiplex kit (ACD, 320850) in RNase-free conditions. To perform the RNAscope *in situ* hybridization, a target retrieval pretreatment protocol was performed as outlined in the manual (ACD, doc. no. 320535). Briefly, slides were submersed in target retrieval buffer (ACD, 322000) at 95–98°C for 10 min, immediately washed in distilled water, dehydrated with 100% ethanol (storage at –80°C if required), and lastly digested with Protease IV for 20 min at 40°C. Following this pretreatment the RNAScope Fluorescent Multiplex Reagent kit User Manual (ACD, doc.no. 320293) was followed and slides were mounted with DAPI-containing Vectashield (Fisher Scientific, NC9029229). A negative control (ACD, 320751) was run in tandem for each experiment. The probes used from ACD Biotechne were as follows: *Crhr1* (418011-C1, -C2), *Gad2* (439371-C3), *Slc17a7* (416631-C2), *Penk* (318761), *Pdyn* (318771), *Sst* (404631), *Npy* (313321), *Prkcd* (441791), *Crh* (316091), and eGFP (400281).

#### Imaging and analysis

Slides were imaged on a Zeiss LSM 780 laser scanning confocal microscope (40× oil immersion, 1024 × 1024, tile scanning of CeA at approximately bregma –1.46 mm, 5-μm z-stacks). All microscope settings were kept the same within experiments during image acquisition. To perform quantification, ImageJ (Schindelin et al., 2012) was used to outline individual nuclei as identified by DAPI staining and count all nuclei in an unbiased manner (all settings kept the same within experiments). The fluorescence intensity for each probe per DAPI counted nuclei was then measured and the background signal, as determined by the average intensity of the negative control, was subtracted for each channel. For each probe, signal intensity present after background subtraction identified positive nuclei. Experiment was performed in an unbiased manner as probe fluorescence was quantified blindly, independently, and after nuclei identification by computational means.

Next, the percentage of nuclei positive for one or both probes and the percentage of signal co-expression were calculated. Percentage of total nuclei was determined by dividing the total number of nuclei expressing that marker by the total number of DAPI positive nuclei per image. The percentage of *Crhr1*+ nuclei expressing a marker of interest was determined by dividing the number of co-labeled nuclei by the total number of *Crhr1*+ nuclei. The percentage of *Crhr1*– nuclei co-expressing a marker of interest was determined by dividing the number of cells expressing the marker of interest but not *Crhr1* by the total number of *Crhr1*– nuclei per image. *Crhr1*+ compared to *Crhr1*– patterns of co-expression was assessed and normalized to the *Crhr1*– cells.

For densitometry, signal intensity measured after background subtraction was quantified, log2 transformed, and normalized to the CeL for visualization of the difference in expression. Unpaired Student’s *t* test was used to assess significance of the CeL versus CeM expression difference for each gene, as well as the *Crhr1*+ to *Crhr1*– percentage co-expression of each gene. Analysis and statistics were performed using R programming (R Core Team, 2018). All analyses were performed on raw images. Outliers were detected by Grubb’s test. Brightness/contrast and pixel dilation are the same for all representative images shown per figures.

### Dendritic spine analysis

#### Tissue preparation

Mice (*n* = 4) were anesthetized (isoflurane) and perfused with cold PBS/4% PFA. Brains were extracted and immersion fixed in 4% PFA at 4°C for 2 h before being sectioned coronally into 100-μm slices on a vibrating microtome (Leica VT1000S, Leica Microsystems).

#### Biolistic labeling

Sections were biolistically labeled with DiI (1,1′-dioctadecyl-3,3,3′,3′-tetramethylindocarbocyanine perchlorate)-coated 1.1-μm tungsten particles delivered with a Bio-Rad gene gun and incubated in PBS overnight at 4°C before immunostaining. Slices were permeabilized in 0.01% Triton X-100 for 15 min at RT with gentle agitation and blocked for 30 min at RT with gentle agitation in blocking solution (10% NGS in 0.01% Triton X-100). Primary antibody incubation was performed overnight at 4°C in PBS (chicken anti-GFP; Abcam, ab13970, RRID:AB_300798; 1:2000). The slices were triple washed with PBS for 10 min followed by secondary antibody incubation for 1 h at RT in blocking solution (Alexa Fluor 488 goat anti-chicken; Thermo Fisher Scientific, A-11039, RRID:AB_142924). The slices were triple washed in PBS and mounted on slides with Prolong Diamond (Thermo Fisher Scientific, P36965).

#### Imaging and analysis

Slides were imaged for CeA on a Zeiss LSM 710 laser scanning confocal microscope (Carl Zeiss MicroImaging; 63× oil immersion, 1024 × 1024, 1-μm step z-stacks). All microscope settings were kept the same within experiments during image acquisition. ImageJ (Schindelin et al., 2012) was used to perform a quantification of filopodia, thin, stubby and mushroom-shaped dendritic spines in both CRF_1_+ (*n* = 13 neurons, 37 dendritic segments) and CRF_1_– neurons (*n* = 24 neurons, 41 dendritic segments). The experimenter was blind to cell type (CRF_1_+ *vs* CRF_1_–) when performing spine quantification. Data obtained in each mouse were averaged for each cell type, such that the number of mice was used as the n in statistical analyses. Spine densities and spine type proportions in CRF_1_– and CRF_1_+ neurons were compared using paired *t* tests. Data are presented as mean ± standard error. In all cases, *p* < 0.05 was the criterion for statistical significance.

### Electrophysiological recordings

#### Brain slice preparation

Mice (*n* = 12) were briefly anesthetized with 3–5% isoflurane and decapitated. Brains were rapidly removed and placed in an ice-cold high-sucrose solution (pH 7.3–7.4) that contained: 206.0 mM sucrose, 2.5 mM KCl, 2.5 mM CaCl_2_, 7 mM MgCl_2_, 1.2 mM NaH_2_PO_4_, 26 mM NaHCO_3_, 5 mM glucose, and 5 mM HEPES. Brains were cut into coronal sections (300 µm) using a Leica 1200s vibratome (Leica Microsystems) and placed in oxygenated (95% O_2_/5% CO_2_) artificial CSF (aCSF) solution composed of the following: 130 mM NaCl, 3.5 mM KCl, 2 mM CaCl_2_, 1.25 mM NaH_2_PO_4_, 24 mM NaHCO_3_, and 10 mM glucose. Slices were incubated in aCSF for 30 min at 37°C, followed by a minimum 30-min equilibration at RT (21–22°C) before use.

#### Electrophysiological recordings

We visualized neurons using infrared differential interference contrast (IR-DIC) optics and CCD camera (Infinity 3 s, Lumenera). A 60× magnification water immersion objective (Olympus) was used for identifying and approaching neurons. To avoid photolytic damage, initial exposure to episcopic fluorescence illumination was brief (<2 s). We detected fluorescent neurons using the Lumen 300 LED system (Prior Scientific) and captured images using Luminera software (Lumenera Corp.). Whole-cell current-clamp and voltage-clamp recordings were obtained with patch pipettes (3–4 MΩ; Warner Instruments) using a Multiclamp 700B amplifier (Molecular Devices), sampled at 20 kHz, low pass filtered at 10 kHz, digitized (Digidata 1440A; Molecular Devices), and stored on a computer using pClamp 10 software (Molecular Devices). Series resistance was not compensated but monitored throughout the experiment, and cells with a series resistance >15 MΩ or a >20% change were excluded.

The intracellular solution used for voltage and current clamp recordings was composed of 145 mM potassium gluconate, 0.5 mM EGTA, 2 mM MgCl_2_, 10 mM HEPES, 2 mM Na-ATP, and 0.2 mM Na-GTP. Drugs were dissolved in aCSF and applied by bath perfusion. To isolate sEPSC and mEPSC, recordings were performed in the presence of GABA_A_ (30 µM bicuculline) and GABA_B_ (1 µM CGP 55845A) receptor antagonists, and tetrodotoxin (TTX; 0.5 µM) was included for mEPSCs. CeA neurons were held at –60 mV for voltage-clamp recordings and maintained at –70 mV for current-clamp recordings.

#### Drugs and chemicals

We purchased CGP 55845A (1 µM) and CRF (200 nM) from Tocris Bioscience, bicuculline methiodide (30 µM) and TTX (0.5 µM) from Sigma.

#### Statistical analysis

Frequency, amplitude, rise, and decay time of EPSCs were analyzed and visually confirmed using a semi-automated threshold based mini detection software (Mini Analysis, Synaptosoft Inc.). Average EPSC characteristics were determined during baseline and experimental drug conditions in 3 min bins containing a minimum of 60 events. All detected events were used for event frequency analysis, but superimposed events were eliminated for amplitude and kinetic analysis. Data are presented as mean ± standard error, and statistical significance was assessed using a two-tailed *t* test or one-sample *t* test using Prism 6 (GraphPad Prism). In all cases, *p* < 0.05 was the criterion for statistical significance, and *n* represents the number of cells or mice as indicated.

## Results

### Expression of CBPs in CeA CRF_1_+ neurons

**Figure 1.**
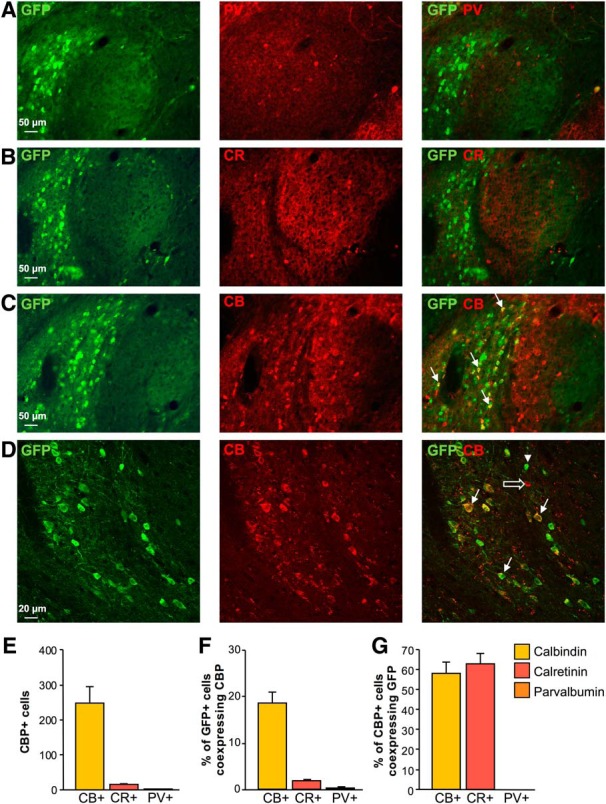
CBP expression in CRF_1_+ cells in the CeA. Representative images of double immunostaining for GFP and (***A***) PV (red, GFP = green), (***B***) CR (red, GFP = green), and (***C***, ***D***) CB (red, GFP = green) in the CeA of CRF_1_:GFP mice. Co-expression patterns are visualized in overlaid images in the third column where the arrowhead indicates a single-labeled GFP+ cell, the open arrow indicates a single-labeled CB+ cell, and filled arrows indicate double-labeled cells that co-express GFP and CB. Scale bars = 50 μm (***A–C***) and 20 μm (***D***). ***E***, Summary bar graph representing the total number of CBP+ cells counted in the CeA. ***F***, Bar graph representing the proportion of CRF_1_+ cells co-expressing each CBP. ***G***, Bar graph representing the proportion of CBP+ cells co-expressing CRF_1_. Data are shown as mean ± SEM. *Figure Contributions*: H. Sidhu performed the experiments and analyzed the data.

The CBPs CB, CR and PV label distinct subsets of neurons in the brain and have been widely used as markers of neuronal populations in the amygdala (Andressen et al., 1993; Kemppainen and Pitkänen, 2000). We therefore examined the expression profile of CBPs in CeA GFP+/CRF_1_+ neurons (Fig. 1). Using immunohistochemistry in CRF_1_:GFP mouse brain sections, the co-expression of PV (PV; Fig. 1*A*), CR (Fig. 1*B*), and CB (Fig. 1*C*,*D*) with GFP was analyzed in the CeA. Of the CBPs labeled, CB was the most widely expressed, while CR was expressed in few cells and PV was virtually absent from the CeA (Fig. 1*E*). The majority of CeA GFP+ cells were located in the CeM, as described previously (Justice et al., 2008; Herman et al., 2013). The percentage of CRF_1_+ cells co-expressing each CBP followed the same pattern as the overall counts of CBP+ cells, wherein CB was present in 19%, CR was present in 2%, and PV was present in 0.1% of CRF_1_+ cells (Fig. 1*F*). Based on GFP labeling in CBP+ cells, CRF_1_ was present in 58% of CB+ cells and 63% of CR+ cells, and no PV+ cells (Fig. 1*G*).

### Expression of neuropeptides and subpopulation markers in CeA *Crhr1*+ neurons

**Figure 2.**
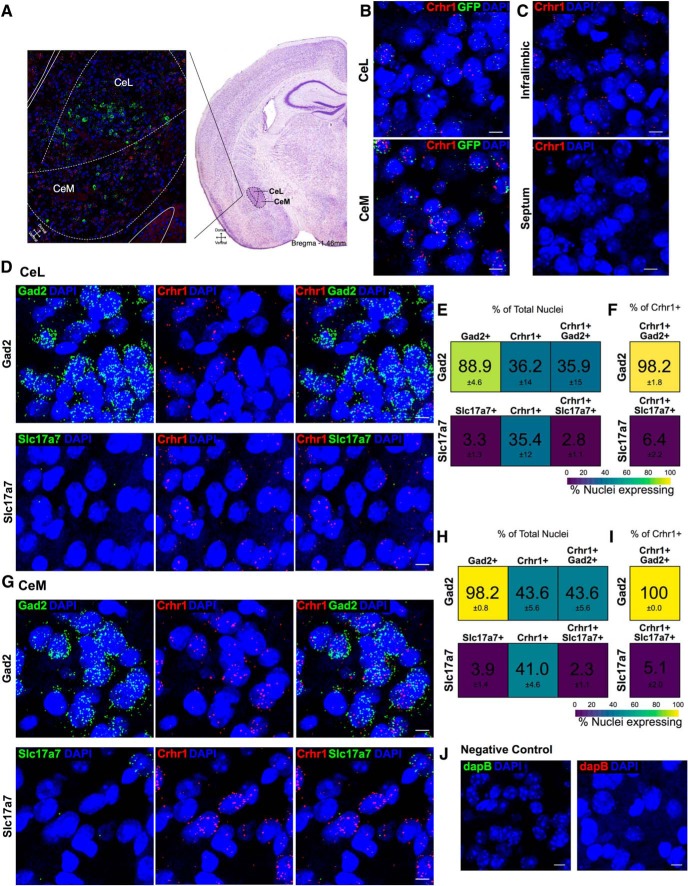
Co-expression of *Gad2* or *Slc17a7* RNA in *Crhr1*+ nuclei. ***A***, Schematic of CeA, with CeL and CeM indicated, is overlaid on tiled image of the CeA in which *Crh* (green), *Crhr1* (red), and DAPI (blue) are shown. Image acquisition position of the CeA (approximately bregma –1.46 mm) is shown on Nissl stained image (Franklin and Paxinos, 2008) to indicate the region of interest. ***B***, Representative images indicate the co-expression of GFP and *Crhr1* in the GFP:CRF_1_ mice in CeL (58%; *n* = 8 images) and CeM (86%; *n* = 7 images; *Crhr1* = red, *GFP* = green, DAPI = blue). Scale bar = 10 μm. ***C***, *Crhr1* probe specificity was validated in the septum of wild-type mice, a region of low *Crhr1*/GFP expression (*n* = 2 images). Negligible expression was observed in the septum as compared to the infralimbic prefrontal cortex in the same slice (*Crhr1* = red, DAPI = blue). Scale bar = 10 μm. ***D***, Representative images for *Gad2* (upper; green = *Gad2*, red = *Crhr1*, blue = DAPI; *n* = 5 images) and *Slc17a7* (lower; green = *Slc17a7*, red = *Crhr1*, blue = DAPI; *n* = 7 images) in the CeL. Scale bar = 10 μm. ***E***, Heat map represents the percentage of nuclei expressing (from left to right) RNA of interest, *Crhr1*, and co-expression in the total nuclei counted in the CeL. ***F***, Heat map represents the percentage of nuclei co-expressing in the *Crhr1+* population of nuclei in the CeL. Color scale from 100% (yellow) to 0% (purple). ***G***, Representative images for *Gad2* (upper; green = *Gad2*, red = *Crhr1*, blue = DAPI; *n* = 5 images) and *Slc17a7* (lower; green = *Slc17a7*, red = *Crhr1*, blue = DAPI; *n* = 7 images) in the CeM. Scale bar = 10 µm. ***H***, Heat map represents the percentage of nuclei expressing (from left to right) RNA of interest, *Crhr1*, and co-expression in the total nuclei counted in the CeM. ***I***, Heat map represents the percentage of nuclei co-expressing in the *Crhr1+* population of nuclei in the CeM. Color scale from 100% (yellow) to 0% (purple). Data are shown as mean ± SEM. ***J***, Representative negative control images for the bacterial probe *DapB* in the CeM indicate negligible fluorescence intensity for both channels shown (*DapB* = red, *DapB* = green, DAPI = blue). Scale bar = 10 μm. *Figure Contributions*: S. A. Wolfe and S. R. D’Ambrosio performed the experiments. S. A. Wolfe analyzed the data.

We next examined the expression of subpopulation markers, including neuropeptides, in *Crhr1*+ neurons in both the CeL and CeM (Fig. 2*A*). First, the validity of the CRF1:GFP transgenic mice was assessed through the co-expression of GFP mRNA and *Crhr1* mRNA in the CeL and CeM where 58% and 86% co-expression in *Crhr1*+ nuclei and 72% and 71% co-expression in GFP mRNA+ nuclei was observed, respectively (Fig. 2*B*). Additionally, the *Crhr1* probe was validated in the septum, a region of low CRF1 expression (Van Pett et al., 2000; Fig. 2*C*). Glutamic acid decarboxylase 2 (*Gad2*) and vesicular glutamate transporter 1 (*Slc17a7*) were used as GABAergic and glutamatergic neuronal markers, respectively. *In situ* hybridization in both the CeL (Fig. 2*D–F*) and CeM (Fig. 2*G–I*) identified a high proportion of nuclei expressing *Gad2*, 89% and 98%, respectively, and a low proportion expressing *Slc17a7*, 3% and 4%, respectively. This is consistent with the majority of neurons being GABAergic in this region (Pitkänen and Amaral, 1994; Ehrlich et al., 2009). Almost all *Crhr1*+ cells expressed *Gad2* at 98% in the CeL and 100% in the CeM (Fig. 2*E*,*H*), whereas very few expressed *Slc17a7* at 6% in the CeL and 5% in the CeM (Fig. 2*F*,*I*). Additionally, a negative control was run in each series for background subtraction (Fig. 2*J*). *Crhr1* expression was observed in ∼ 23% and 34% of all nuclei on average in CeL and CeM, respectively. These data indicate that CeA *Crhr1+* neurons, as well as the vast majority of CeA neurons, are GABAergic and are located in both the CeL and CeM.

**Figure 3.**
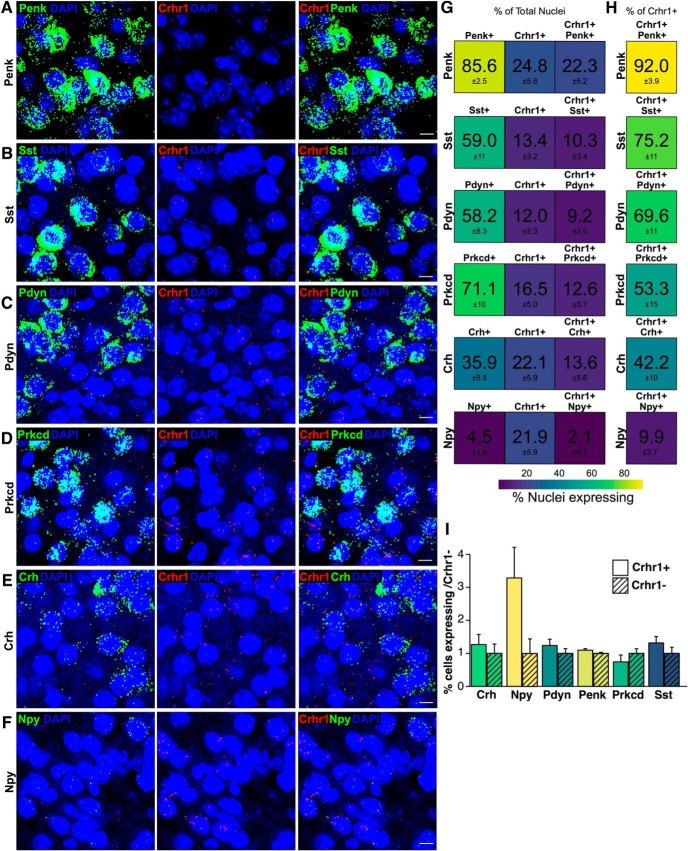
Co-expression of RNA of interest in *Crhr1*+ nuclei in the CeL. Representative images from top to bottom for (***A***) *Penk* (green *= Penk*, red = *Crhr1*, blue = DAPI; *n* = 8 images), (***B***) *Sst* (green = *Sst*, red = *Crhr1*, blue = DAPI; *n* = 9 images), (***C***) *Pdyn* (green = *Pdyn*, red = *Crhr1*, blue = DAPI; *n* = 10 images), (***D***) *Prkcd* (green = *Prkcd*, red = *Crhr1*, blue = DAPI; *n* = 9 images), (***E***) *Crh* (green = *Crh*, red = *Crhr1*, blue = DAPI; *n* = 10 images), and (***F***) *Npy* (green = *Npy*, red = *Crhr1*, blue = DAPI; *n* = 9 images) in the CeL. Scale bar = 10 μm. ***G***, Heat map represents the percentage of nuclei expressing (from left to right) RNA of interest, *Crhr1*, and co-expression in the total nuclei counted in the CeM. ***H***, Heat map represents the percentage of nuclei co-expressing in the *Crhr1+* population of nuclei in the CeM. Color scale from 100% (yellow) to 0% (purple). ***I***, Quantification of the difference between the percentage nuclei expressing RNA of interest (*Crh*, *Prkcd*, *Pdyn*, *Npy*, *Penk*, and *Sst*) in *Crhr1*+ nuclei versus *Crhr1*– nuclei in the CeL (mean ± SEM). *Figure Contributions*: S. A. Wolfe performed the experiments and analyzed the data.

**Figure 4.**
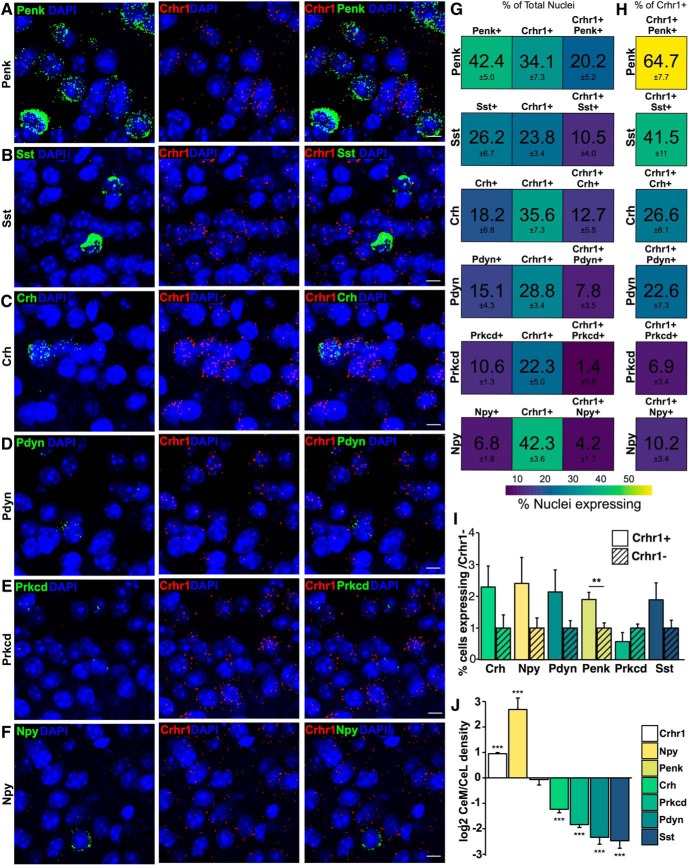
Co-expression of RNA of interest in *Crhr1*+ nuclei in the CeM. Representative images from top to bottom for (***A***) *Penk* (green = *Penk*, red = *Crhr1*, blue = DAPI; *n* = 11 images), (***B***) *Sst* (green = *Sst*, red = *Crhr1*, blue = DAPI; *n* = 10 images), (***C***) *Crh* (green = *Crh*, red = *Crhr1*, blue = DAPI; *n* = 11 images), (***D***) *Pdyn* (green = *Pdyn*, red = *Crhr1*, blue = DAPI; *n* = 8 images), (***E***) *Prkcd* (green = *Prkcd*, red = *Crhr1*, blue = DAPI; *n* = 6 images), and (***F***) *Npy* (green = *Npy*, red = *Crhr1*, blue = DAPI; *n* = 8 images) in the CeM. ***G***, Heat map represents the percentage of nuclei expressing (from left to right) RNA of interest, *Crhr1*, and co-expression in the total nuclei counted in the CeM. ***H***, Heat map represents the percentage of nuclei co-expressing in the *Crhr1+* population of nuclei in the CeM. Color scale from 60% (yellow) to 0% (purple). ***I***, Quantification of the difference between the percentage nuclei expressing RNA of interest [*Crh*, *Prkcd*, *Pdyn*, *Npy*, *Penk* (*p* = 0.004), and *Sst*] in *Crhr1*+ nuclei versus *Crhr1*– nuclei in the CeM (mean ± SEM; **p < 0.01 unpaired *t* test). ***J***, Bar graph showing the difference in log2 of the signal intensity between CeL and CeM of *Crhr1* (*n* = 3079 nuclei), *Npy* (*n* = 90 nuclei), *Penk* (*n* = 468 nuclei), *Crh* (*n* = 273 nuclei), *Prkcd* (*n* = 276 nuclei), Pdyn (*n* = 141 nuclei), and *Sst* (*n* = 145 nuclei) in *Crhr1*+ nuclei (mean ± SEM; ***p < 0.001 unpaired *t* test from CeL). *Figure Contributions*: S. A. Wolfe performed the experiments and analyzed the data.

Co-expression of *Crhr1* with the neuropeptides *Penk*, *Sst*, *Pdyn*, *Crh*, and *Npy*, as well as with *Prkcd*, was also examined (CeL, Fig. 3*A–F*; CeM, Fig. 4*A–F*). In the CeL, transcripts for all these genes, except *Npy*, were found in high abundance, where *Penk* was expressed in 86% of cells, *Sst* in 59%, *Pdyn* in 58%, *Prkcd* in 71%, *Crh* in 36%, and *Npy* in 5% of cells (Fig. 3*G*). The proportion of total nuclei expressing each of these transcripts was similar to the proportion observed in *Crhr1*+ nuclei only, where *Penk* was expressed in 92%, *Sst* in 75%, *Pdyn* in 70%, *Prkcd* in 53%, *Crh* in 42%, and *Npy* in 10% of *Crhr1*+ cells (Fig. 3*H*). Accordingly, when comparing the expression of the above peptides in CeL *Crhr1*+ versus *Crhr1*– neurons no significant differences were observed between the two populations (Fig. 3*I*).

The CeM showed a similar general co-expression pattern as the CeL (Fig. 4*A–F*). However, the proportion of total nuclei expressing neuropeptides was overall lower, with the percentage expressing *Penk* at 42%, *Sst* at 26%, *Crh* at 18%, *Pdyn* at 15%, *Prkcd* at 11%, and *Npy* at 7% (Fig. 4*G*). Similar proportions were observed in *Crhr1*+ nuclei only, with the percentage expressing *Penk* at 65%, *Sst* at 42%, *Crh* at 27%, *Pdyn* at 23%, *Prkcd* at 7%, and *Npy* at 10% (Fig. 4*H*). *Penk* and *Sst* again presented the strongest co-expression and *Npy* the weakest. When comparing the expression of the above peptides in CeM *Crhr1*+ versus *Crhr1*– neurons, *Penk* was found significantly enriched in *Crhr1*+ neurons (1.9-fold, *p* = 0.004; Fig. 4*I*). Lastly, we compared the density of nuclear expression of these co-expressed genes in CeM compared to CeL *Crhr1*+ nuclei (Fig. 4*J*), and found that the CeM expressed significantly lower amounts of RNA for all targets [*Crh* (–1.2-fold, *p* = 1.2e-7), *Prkcd* (–1.84-fold, *p* = 1.3e-14), *Pdyn* (–2.3-fold, *p* = 8.4e-11), and *Sst* (–2.95-fold, *p* = 3.8e-10)] except *Penk*, which was expressed at equivalent levels in CeM and CeL (–0.07-fold), and *Npy* (2.7-fold, *p* = 2.5e-7), whose mRNA density was higher in CeM. Additionally, we observed that *Crhr1* density was higher in the CeM compared to the CeL (*p* = 2.2e-16; Fig. 4*J*).

### Dendritic spine morphology and density in CRF_1_+ and CRF_1_– CeM neurons

**Figure 5.**
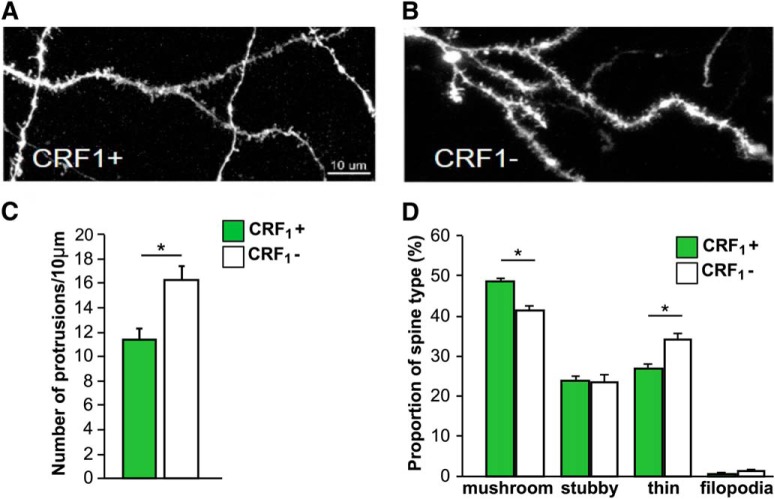
Spine density and morphology of CRF_1_+ and CRF_1_– neurons in the CeA. Representative images of dendritic segments in CRF_1_+ (***A***) and CRF_1_– (***B***) neurons from the CeM of CRF_1_:GFP male mice. Scale bar = 10 µm. ***C***, Summary bar graph indicates spine density in CRF_1_+ and CRF_1_– neurons averaged over 10 µm dendritic segments (*p < 0.05 paired *t* test). ***D***, Summary graph indicates proportion of mushroom spines, stubby spines, thin spines and filopodia in CRF_1_+ and CRF_1_– neurons in the CeA (*p < 0.05, paired *t* test). Data are shown as mean ± SEM (*n* = 4 mice). *Figure Contributions*: H. Sidhu performed the experiments. H. Sidhu and C. Contet analyzed the data.

Higher levels of GFP and *Crhr1* mRNA expression were observed in the CeM than in the CeL (Figs. 1–4), as previously reported (Justice et al., 2008; Herman et al., 2013). We therefore focused subsequent analyses of CRF_1_+ and CRF_1_– neurons in the CeM. We first analyzed dendritic spines using biolistic labeling of neurons with a lipophilic fluorescent dye followed by GFP immunostaining (Fig. 5*A*,*B*). Spine density in CRF_1_+ (11 spines/10 μm) neurons was lower than in CRF_1_– (16 spines/10 μm) neurons (paired *t* test, *t*_(3)_ = –3.3, *p* < 0.05; Fig. 5*C*). We further analyzed the morphology of dendritic protrusions and calculated the proportion of mushroom spines, stubby spines, thin spines and filopodia in CRF_1_+ (49%, 24%, 27%, and 0.7%, respectively) and CRF_1_– neurons (41%, 23%, 34%, and 1.2%, respectively; Fig. 5*D*). The proportion of mushroom spines was significantly higher (*t*_(3)_ = 4.9, *p* < 0.05) while the proportion of thin spines was significantly lower (*t*_(3)_ = –4.9, *p* < 0.05) in CRF_1_+ neurons compared to CRF_1_– neurons. There were no differences in the proportion of stubby spines (*t*_(3)_ = 0.4, n.s.) and filopodia (*t*_(3)_ = –1.0, n.s.) between the two cell types.

### Glutamatergic transmission in CRF_1_+ and CRF_1_– neurons in the CeM

**Figure 6.**
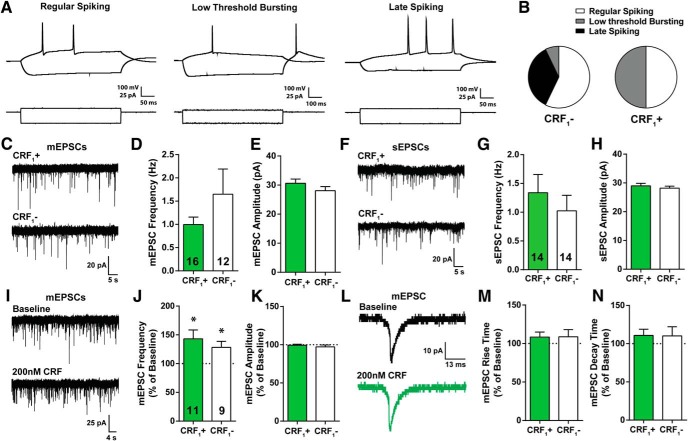
Membrane properties and glutamatergic transmission of CRF_1_+ and CRF_1_– neurons in the CeA. ***A***, Representative current-clamp recordings from three cell-types: regular spiking (left), low threshold bursting (middle) and late spiking (right) from a CeA neuron. ***B***, Distribution of cell-types recorded from for CRF_1_– (*n* = 15) and CRF_1_+ (*n* = 16) CeA neurons. ***C***, Representative traces of mEPSCs in CRF_1_+ (top) and CRF_1_– (bottom) CeA neurons. ***D***, ***E***, Frequency and amplitude of mEPSCs in CRF_1_+ (*n* = 16) and CRF_1_– (*n* = 12) CeA neurons. ***F***, Representative traces of sEPSCs in CRF_1_+ (top) and CRF_1_– (bottom) CeA neurons. ***G***, ***H***, Frequency and amplitude of sEPSCs in CRF_1_+ (*n* = 14) and CRF_1_– (*n* = 14) CeA neurons. ***I***, Representative traces of mEPCS during baseline (top) and 200 nM CRF application (bottom) from CRF_1_+ CeA neurons. ***J***, ***K***, CRF effects on frequency and amplitude of mEPSCs in CRF_1_+ (*n* = 11) and CRF_1_– (*n* = 9) CeA neurons, respectively. ***L***, Average trace of mEPSC during baseline (black) and 200 nM CRF (green) application from a CRF_1_+ CeA neuron. ***M***, ***N***, CRF effects on rise and decay time of mEPSCs in CRF_1_+ and CRF_1_– CeA neurons, respectively (*p < 0.05 by one-sample *t* test). Data are shown as mean ± SEM. *Figure Contributions*: R. R. Patel performed the experiments and analyzed the data.

We next examined glutamatergic transmission in CRF_1_+ and CRF_1_– CeM neurons. Before recording glutamatergic activity, whole-cell current clamp recordings with a step protocol consisting of hyperpolarizing to depolarizing current injections were obtained from each cell to determine cell type based on spiking characteristics. As previously described, CeA neurons are composed of three principal cell types: regular spiking, low threshold bursting, and late spiking neurons (Dumont et al., 2002; Chieng et al., 2006; Herman et al., 2013; Fig. 6*A*). CRF_1_+ neurons consisted of only regular spiking and low threshold bursting cell-types as previously reported (Herman et al., 2013, 2016), while the majority of CRF_1_– neurons were regular spiking and late spiking neurons (Fig. 6*B*). No significant differences were observed in the membrane properties of CRF_1_+ and CRF_1_– CeA neurons (Table 1).

**Table 1.** Membrane properties of recorded CeA neurons

	Membrane capacitance (pF)	Membrane resistance (MΩ)	Time constant (ms)	Resting membrane potential (mV)
*CRF_1_*+	44.36 ± 2.7	476.0 ± 34.8	369.2 ± 52.4	–52.1 ± 1.8
*CRF_1_*–	49.91 ± 2.9	456.2 ± 13.3	348.4 ± 55.5	–52.6 ± 1.8

The membrane capacitance, resistance, time constant, and resting potential are reported for CRF1+ and CRF1– neurons recorded from the CeA. Data are shown as mean ± SEM. *Table Contributions*: R. R. Patel performed experiments and analyzed data.

We then assessed baseline glutamatergic transmission using whole-cell voltage clamp recordings of spontaneous action-potential dependent and miniature action-potential independent EPSCs (sEPSCs and mEPSCs, respectively) in CRF_1_+ and CRF_1_– CeM neurons (Fig. 6*C–H*). We observed similar baseline mEPSC frequency (*t*_(26)_ = 1.3, n.s.) and amplitude (*t*_(26)_ = 1.23, n.s.) in CRF_1_+ (0.9 ± 0.16 Hz; 30.6 ± 1.4 pA; *n* = 16) and CRF_1_– neurons (1.6 ± 0.5 Hz; 28.0 ± 1.4 pA; *n* = 12; Fig. 6*C–E*), and there were no differences in rise and decay kinetics of mEPSCs (Table 2). We also observed similar baseline sEPSC frequency (*t*_(26)_ = 0.75, n.s.) and amplitude (*t*_(26)_ = 0.70, n.s.) in CRF_1_+ (1.3 ± 0.3 Hz; 28.9 ± 0.8 pA; *n* = 14) and CRF_1_– neurons (1.0 ± 0.2 Hz; 28.1 ± 0.6 pA; *n* = 14; Fig. 6*F–H*), and there were no differences in rise and decay kinetics of sEPSCs (Table 2). Overall, these two populations receive similar glutamatergic input, consistent with their similar density of mature spines.

**Table 2.** Summary of mEPSC and sEPSC characteristics

	Frequency(Hz)	Amplitude(pA)	Rise time(ms)	Decay time(ms)
*mEPSCs*				
CRF_1_–	1.64 ± 0.54	28.09 ± 1.40	0.95 ± 0.14	0.56 ± 0.10
CRF_1_+	0.99 ± 0.16	30.63 ± 1.43	1.02 ± 0.06	0.56 ± 0.06
*sEPSCs*				
CRF_1_–	1.02 ± 0.26	28.18 ± 0.68	0.84 ± 0.07	0.60 ± 0.07
CRF_1_+	1.33 ± 0.31	28.96 ± 0.87	0.90 ± 0.10	0.62 ± 0.08

The frequency, amplitude, rise time, and decay time of mEPSCs and sEPSCs are reported for CRF1+ and CRF1– neurons recorded from the CeA. Data are shown as mean ± SEM. *Table Contributions*: R. R. Patel performed experiments and analyzed data.

### CRF effects on glutamatergic signaling in CRF_1_+ and CRF_1_– CeM neurons

To assess differences in the functional responsivity of CRF_1_+ and CRF_1_– populations to CRF, we tested the effect of CRF (200 nM; 9–12 min) on mEPSCs (Fig. 6*I–N*). We found that CRF application increased mEPSC frequency in both CRF_1_+ (*t*_(10)_ = 2.78, *p* < 0.05) and CRF_1_– (*t*_(8)_ = 2.68, *p* < 0.05) CeM neurons (Fig. 6*J*) but did not significantly alter mEPSC amplitude (CRF_1_+: *t*_(10)_ = 0.50, n.s.; CRF_1_–: *t*_(8)_ = 1.12, n.s.; Fig. 6*K*), rise time (CRF_1_+: *t*_(10)_ = 1.28, n.s.; CRF_1_–: *t*_(8)_ = 0.95, n.s.; Fig. 6*M*), and decay time (CRF1+: *t*_(10)_ = 1.32, n.s.; CRF_1_–: *t*_(8)_ = 0.85, n.s.; Fig. 6*N*) of mEPSCs in either population (Fig. 6). In summary, CRF globally increases glutamatergic transmission in the CeM via increased presynaptic GABA release, but does not alter mEPSC amplitude or kinetics, suggesting a lack of postsynaptic effect of CRF in either population.

## Discussion

Here, we investigated the neurochemistry, morphology, and physiology of CRF_1_+ neurons in the CeA of adult male mice. We found that CB is the predominant CBP expressed in the CeA but overall there is no specific enrichment or exclusion of CBPs in *Crhr1+* neurons. Co-expression analysis using *in situ* hybridization revealed *Crhr1* is co-expressed mostly with *Penk* and *Sst* and least with *Npy*. In the CeM, *Penk* is significantly enriched in CRF_1_+ neurons compared to CRF_1_– neurons. Morphologically, CRF_1_+ neurons have a lower spine density but a higher proportion of mushroom spines compared to CRF_1_– neurons. Accordingly, basal excitatory transmission between CRF_1_+ and CRF_1_– neurons are similar. Additionally, acute CRF application increases glutamatergic transmission in both CRF_1_+ and CRF_1_– neurons. The lack of enrichment or exclusion of any particular CBP or neuropeptide in this population contributes to the difficulty in identifying and studying this subpopulation in this heterogenous region.

### CBP and neuropeptide expression in the CRF_1_+ subpopulation

GABAergic interneurons may be subcategorized based on their expression of the CBPs PV, CB, and CR, and these distinct subpopulations exhibit unique differences in their physiology, synaptology, and morphology (Kemppainen and Pitkänen, 2000). We found that CB was the predominant CBP in both CeL and CeM nuclei, with only a limited number of neurons expressing PV and CR. This pattern of relative CBP expression is in general agreement with immunohistochemical studies conducted in the rat (Kemppainen and Pitkänen, 2000), and with our previous finding that at least 30% of CeA CRF_1_–expressing neurons project out to the dorsolateral BNST (Herman et al., 2013, 2016), one of several downstream brain regions innervated by the CeA.

In addition, CeA neurons can express several neuropeptides that play important roles in fear and anxiety behaviors such as CRF, enkephalins, dynorphins, somatostatin and NPY. The overlapping expression pattern of these neuropeptides differentiates distinct neuronal subpopulations in several brain regions in mice and rats (Petrovich et al., 1996; Herry et al., 2008; Haubensak et al., 2010; Li et al., 2013; Cai et al., 2014; Penzo et al., 2014; Yu et al., 2016; Kim et al., 2017). We hypothesized that CRF_1_ expression is restricted to a subset of CeA neurons that co-express a unique combination of neuropeptides. Most of the neuropeptides we investigated had similar trends in the total population compared to the *Crhr1*+ population. However, *Penk* was present in a higher proportion of *Crhr1*+ cells than *Crhr1*– cells in the CeM. *Npy* also trended toward an enrichment in *Crhr1*+ cells over *Crhr1*– cells in the CeL but co-expression results obtained for this neuropeptide showed more variability than for other markers, most likely due to the small number of *Npy*+ nuclei. Additionally, densitometry identified a higher expression of *Crh*, *Pdyn*, *Prkcd*, and *Sst* in the CeL compared to the CeM, which is in agreement with previous studies in mice and rats (Veinante et al., 1997; Day et al., 1999; Marchant et al., 2007; Li et al., 2013; Kim et al., 2017; McCullough et al., 2018). Conversely, *Npy* was present at higher levels in the CeM than in the CeL.

The enrichment of *Penk* in CeM *Crhr1*+ neurons compared to their *Crhr1*– neighbors may have functional relevance for the recruitment of CeA *Penk*+ neurons in response to food, drugs and stress. CeA *Penk*+ are activated by subchronic exposure to fat, ethanol, and nicotine, as well as by withdrawal from morphine in animal models (Criado and Morales, 2000; Veinante et al., 2003; Loughlin et al., 2006; Chang et al., 2014). This activation of CeA *Penk*+ neurons may result from their preferential expression of CRF_1_ as some of these stimuli are known to increase CRF_1_ signaling in the CeA (Heinrichs et al., 1995; Nie et al., 2004). Furthermore, chemogenetic activation of CeA *Penk* neurons produces sustained analgesia, suggesting that CRF_1_-mediated activation of these neurons could mediate stress-induced analgesia (Wiedenmayer et al., 2002; Paretkar and Dimitrov, 2019).

It is important to consider here that our method for identification of expressing cells is semi-quantitative and that only signal co-localized with DAPI staining after stringent background subtraction was quantified to increase confidence in detection. However, by excluding cell bodies and processes, we lost data concerning dynamic somatic and neurite expression. This method is also limited in its ability to distinguish only neuronal cells which may introduce a consistent level of non-neuronal cells in our analysis. Additionally, co-expression may vary from the rostral to caudal CeA, and a more in-depth study of CeA neuropeptide localization is necessary to identify the role of these neuropeptides in the CRF_1_+ subpopulation throughout the CeA. Further studies to identify dynamic expression of RNA or protein at subcellular levels may yield additional information concerning the functional roles of these peptides and proteins.

Our *in situ* hybridization analysis of *Crhr1* expression may yield differing results than GFP expression in the CRF_1_:GFP mice due to differences in RNA versus protein expression, sensitivity of the methods used, image acquisition, and identification of GFP saturated soma versus puncta quantification in DAPI-stained nuclei. However, the *Crhr1* probe was verified to yield negligible signal in the septum, a region with no CRF_1_/GFP expression (Van Pett et al., 2000). Furthermore, our observation that *Crhr1* mRNA density is markedly higher in the CeM than in the CeL is consistent with the patterns of GFP native fluorescence and immunohistochemical staining reported here (Fig. 2*B*) and previously (Justice et al., 2008; Herman et al., 2013), with GFP expression being substantially more prominent in the CeM than in the CeL. Additionally, probe sensitivity and mRNA abundance might affect the analysis of *Crhr1* and GFP mRNA co-expression. A strong overlap was found between both populations in the CeM, as well as a strong co-expression in GFP mRNA+ nuclei in the CeL. However, co-expression was less pronounced in *Crhr1*+ nuclei in the CeL, reflective of less GFP protein expression (Fig. 2*B*). The higher sensitivity of the RNAscope assay compared to native fluorescence and immunohistochemistry probably explains why ∼23% CeL cells are detected as *Crhr1*+, while a very limited number of CeL cells are identified as GFP+.

### CRF_1_+ neurons differ from CRF_1_– neurons in their dendritic morphology

We characterized the dendritic morphology of CeA CRF_1_+ and CRF_1_– neurons including spine density and spine shape. The morphology and density of spines plays a crucial role in synaptic and neuronal function. More mature and enlarged spines (mushroom/stubby) are associated with increased synaptic strength, whereas immature spines (thin/filopodia) may sustain limited synaptic signaling (De Roo et al., 2008; Sala and Segal, 2014). Abnormalities in spine morphology are associated with a variety of neurologic and psychiatric disorders, including addiction (Mulholland and Chandler, 2007; Spiga et al., 2014; Phillips and Pozzo-Miller, 2015; Herms and Dorostkar, 2016; Qiao et al., 2016; Varodayan et al., 2018). Previous morphologic characterization of CeA neurons has identified different cell types (McDonald, 1982; Chieng et al., 2006). Most neurons in the CeM have long dendrites that branch sparingly and have a moderate number of dendritic spines, while a smaller number of neurons have thick dendrites with virtually no spines (McDonald, 1982). Here, we observed that the spine density in CRF_1_+ neurons was lower than in CRF_1_– neurons. We further analyzed the spine type proportions in CRF_1_+ and CRF_1_– neurons and found a higher proportion of mushroom spines and lower proportion of thin spines in CRF_1_+ neurons. The lower overall spine density is therefore at least partially offset by a higher fraction of mushroom (i.e., most mature) spines, resulting in a comparable density of functional spines in CRF_1_+ and CRF_1_– neurons.

### CRF modulation of glutamatergic transmission in CRF_1_+ versus CRF_1_– neurons

CRF modulates glutamate transmission and has been shown to increase vesicular glutamate release at rat CeA synapses, which can be enhanced by CRF_1_ activity (Varodayan et al., 2017). Therefore, we examined glutamatergic transmission in CRF_1_+ and CRF_1_– cells as well as the effect of CRF on glutamatergic transmission in these neuronal populations. Both populations receive similar glutamatergic input, consistent with the similar density of functional spines in CRF_1_+ and CRF_1_– neurons. Additionally, CRF increases mEPSC frequency in both populations with no effect on mEPSC amplitude or kinetics in either population. These results are indicative of CRF-induced increased presynaptic GABA release with no postsynaptic effect on glutamatergic receptors.

## Conclusion

Our results reveal molecular, morphologic and functional characteristics of CRF_1_+ neurons highlighting the importance of identifying specific cell populations in the CeA. The CeA is a hub that integrates disparate inputs (stress) and drives appropriate behavioral outputs. Previous work has demonstrated a major role of the CRF system, and particularly the CRF_1_ receptors, within the amygdala complex to influence cellular functions that produce maladaptive behavior in animal models. The amygdalar CRF_1_ system represents a common pathway for the convergence of stress, addiction, pain, depression, memory formation and anxiety-related signaling (Koob, 2003, 2008; Zorrilla and Koob, 2004). In particular, CRF_1_ mechanisms and circuits have been implicated in the development of the negative emotional state associated with alcohol dependence, and it has been proposed that alleviation of this negative state drives the motivation to drink in mice and rats (Funk et al., 2006; Koob, 2010; Roberto et al., 2010; Lowery-Gionta et al., 2012). Our cellular studies revealed that acute and chronic alcohol induced significant alterations in GABAergic signaling in the CeA circuitry. In addition, this GABAergic signaling is cell type specific for the CRF_1_+ subpopulation and their connectivity and may contribute to the development of alcohol dependence (Herman et al., 2016).

Our new evidence on the CRF_1_+ cellular phenotype details the distinct neuronal CeA subpopulations in normal amygdalar function and highlights the need for their further characterization (including intracellular signaling etc) under pathologic conditions, such as alcohol dependence and anxiety disorders. The multiplicity of neuronal subpopulations and the complexity of local microcircuits provide numerous targets for potential dysregulation by drugs of abuse (e.g., alcohol) and stress, and their potential clinical relevance.

## Synthesis

Reviewing Editor: Yavin Shaham, NIDA-IRP/NIH Decisions are customarily a result of the Reviewing Editor and the peer reviewers coming together and discussing their recommendations until a consensus is reached. When revisions are invited, a fact-based synthesis statement explaining their decision and outlining what is needed to prepare a revision will be listed below. The following reviewer(s) agreed to reveal their identity: Mario Penzo.

The reviewers found merit in the study but also have raised several significant concerns that should be addressed in the revision. It is particularly important to satisfactory address comments 1 and 4 of Reviewer 1. I enclose below the individual comments of the reviewers and below each of them my recommendation of how to address those. In addition to their comments, please also address the comments below:

1. Significance and Introduction sections: change 'numerous' to 'several'.

2. Introduction and Discussion section: when referring to the role of CRF in psychiatric disorders, please qualify the statement to indicate that this is in reference to animal models of psychiatric disorders, not human psychiatric disorders. Also, somewhere in one of these sections, the journal's readers should be aware that clinical studies derived from the preclinical animal model studies were unsuccessful (Heilig alcohol studies, Nemeroff-Mayberg PTSD study [PMID:28793974] and other negative clinical studies on the lack of effect of CRF1 antagonist on depression and anxiety).

-3. Methods: please report whether the cell counts were done in a blind manner.

-4. Results section, dendritic spine data analysis: from the degrees of freedom of the t-tests, it appears that the data were analyzed using the dendrite as the n; this violates assumptions of parametric statistics that require independence of observations within a sample. For the revision, these data should be analyzed using the mouse, not the dendrite, as the n in the analysis.

-5. Discussion: please add 2-3 informative sub-headings of the topics being discussed.

REVIEWER 1

The manuscript entitled “Molecular, morphological and functional characterization of corticotropin-releasing factor 1-expressing neurons in the central nucleus of the amygdala” provides a useful but rather basic description of CRF1+ cells of the CeA. While the authors have mostly successfully implemented immunohistochemical and in situ hybridization methods to molecularly define CRF1+ neurons, their efforts to address the “morphological” and “functional” portions of the manuscript's title leave something to be desired. Indeed, the “morphological” criterion is attempted to be fulfilled with limited spine quantification and the functional part is addressed with hard-to-interpret electrophysiological data. Thus, in its current form the manuscript is rather thin. To be considered for

publication the authors should address the following.

1) It is well known that BAC transgenics often do not recapitulate the full endogenous pattern of gene expression. The authors should use in situ hybridization to provide experimental evidence of the fidelity of this transgenic line.

Editor: I recommend adding these data as these are critical to the interpretation of the results in the paper.

2) The distribution of genetically-defined classes of CeA neurons have been previously shown to vary across the rostro-caudal axis of the amygdala. The authors should assess whether co-expression of Crhr1 and known markers of CeA neurons (SSt, Prkc, Pdyn, Enk... etc) varies rostro-caudally.

Editor: These data will strengthen the paper but they are not mandatory for a successful revision. This anatomical issue should be addressed with text changes in the revision.

3) Neurons of the CeM are basically considered the output cells of the amygdala circuit and innervate various targets such as the hypothalamus and the periaqueductal grey. Considering the scope of the present study, it would be interesting if the characterization performed here also included an evaluation of the downstream targets of CRF1+ neurons. Such investigation would help satisfy the need for more comprehensive morphological/anatomical data here. At the very least the authors could use their patch clamp experiments as an opportunity to fill and reconstruct individual CRF1+ and CRF1- neurons.

Editor: As with point 2, these data will strengthen the paper but they are not mandatory for a successful revision. These anatomical and functional issues should be addressed with text changes in the revision.

4) The electrophysiological portion of this study is quite weak. For instance, it is unclear why the authors assessed the effect of CRF (200nM) on spontaneous transmission onto both CRF1+ and - cells. Because changes in the frequency and/or amplitude of sEPSCs can result from network effects (non-synaptic), it is difficult to draw any useful conclusions from this experiment. The authors should repeat this experiment while measuring mEPSCs. Similarly, and to get a more complete picture, the authors should also investigate the effect of CRF on inhibitory synaptic transmission (mIPSC) in CeM neurons (both CRF1+ and -). This is of particular interest, considering that the CeL/CeM form an inhibitory circuit. Also, since most CRF1+ neurons are in the CeM and PKCd+ neurons of the CeL are thought to provide most of CeL's output to CeM, the authors could cross CRF1-GFP mice with PKCd-Cre and investigate the effect of CRF on synaptic inhibition in the CeL-CeM pathway. This would significantly improve the quality of the manuscript.

Editor: After a consultation with the reviewers, the consensus was that the authors should add mEPSC measurements instead of or in addition to sEPSC, as sEPSC changes could be due to network changes and not necessarily synaptic ones.

Minor:

1) Figure 6 is simply reproducing previously described results. Because this is essentially proof of principle, there is no need to include these data in a separate figure.

Editor: please follow the reviewer's recommendation.

REVIEWER 2

This manuscript provides a descriptive characterization of the CRF1-expressing neurons in the CeA and compares them with the CeA cells that do not express CRF1. The authors evaluate the colocalization of CRF1 with other molecules in the CeA and also provide a description of the morphological and electrophysiological properties of these cells. The results presented are new and confirm that cells in the CeA, even those expressing a specific molecule, are highly heterogenous. These results are important and should be taken into consideration when studying this population of cells in the CeA. The function of these cells in the modulation of behaviors was not addressed. Given that the results are entirely descriptive and do not identify a robust feature that distinguishes these cells from all other cells in the CeA, I think that the advancement is important but somewhat incremental.

Editor: please address the reviewer's comments with text changes in the revision.
